# Improving rice blast resistance of Feng39S through molecular marker-assisted backcrossing

**DOI:** 10.1186/s12284-019-0329-3

**Published:** 2019-09-09

**Authors:** Dabing Yang, Jianhao Tang, Di Yang, Ying Chen, Jauhar Ali, Tongmin Mou

**Affiliations:** 1 National Key Laboratory of Crop Genetic Improvement and National Centre of Plant Gene Research (Wuhan), Huazhong Agricultural University, Wuhan, China; 2 Rice Breeding Platform, International Rice Research Institute, DAPO Box 7777, Metro Manila, Philippines

**Keywords:** PTGMS line, Two-line hybrid rice, Rice blast resistance, Marker-assisted background selection, Genomics-based breeding

## Abstract

**Background:**

Rice blast caused by *Magnaporthe oryzae* is one of the most widespread biotic constraints that threaten rice production. Using major resistance genes for rice blast resistance improvement is considered to be an efficient and technically feasible approach to achieve optimal grain yield.

**Results:**

We report here the introgression of the broad-spectrum blast resistance gene *Pi2* into the genetic background of an elite PTGMS line, Feng39S, for enhancing it and its derived hybrid blast resistance through marker-assisted backcrossing (MABC) coupled with genomics-based background selection. Two PTGMS lines, designated as DB16206–34 and DB16206–38, stacking homozygous *Pi2* were selected, and their genetic background had recurrent parent genome recovery of 99.67% detected by the SNP array RICE6K. DB16206–34 and DB16206–38 had high resistance frequency, with an average of 94.7%, when infected with 57 blast isolates over 2 years, and the resistance frequency of their derived hybrids ranged from 68.2% to 95.5% under inoculation of 22 blast isolates. The evaluation of results under natural blast epidemic field conditions showed that the selected PTGMS lines and their derived hybrids were resistant against leaf and neck blast. The characterizations of the critical temperature point of fertility-sterility alternation of the selected PTGMS lines, yield, main agronomic traits, and rice quality of the selected PTGMS lines and their hybrids were identical to those of the recurrent parent and its hybrids. DB16206–34/9311 or DB16206–38/9311 can be used as a blast-resistant version to replace the popular hybrid Fengliangyou 4. Likewise, DB16206–34/FXH No.1 or DB16206–38/FXH No.1 can also be used as a blast-resistant version to replace another popular hybrid Fengliangyou Xiang 1.

**Conclusions:**

Our evaluation is the first successful case to apply MABC with genomics-based background selection to improve the blast resistance of PTGMS lines for two-line hybrid rice breeding.

**Electronic supplementary material:**

The online version of this article (10.1186/s12284-019-0329-3) contains supplementary material, which is available to authorized users.

## Background

Rice (*Oryza sativa* L.) is one of the most important staple food crops, supplying calories for more than half of the world’s population. Rice blast, caused by *Magnaporthe oryzae* (*M. oryzae*), is one of the most important fungal diseases. Yield losses due to blast were reported to be from 30% to 50% in large rice planting areas under favorable environmental conditions (Skamnioti and Gurr, 2009), while the annual loss of rice production caused by blast could fulfill the annual rice consumption of 60 million people. Fatal leaf and neck blast could especially result in severe yield losses and even zero production (Parker et al. 2008). Tremendous efforts have been devoted to evaluating and characterizing blast resistance genes, and more than 70 resistance genes and QTLs against *M. oryzae* have been identified and mapped (Kou and Wang, 2012). To date, at least 25 rice blast R genes have been cloned and characterized (Luo et al. 2017a). Among them, the *Pi2* locus confers broad-spectrum resistance to various *M. oryzae* isolates (Liu et al. 2002). Several attempts had been made to deploy *Pi2* in rice breeding, including improvement for maintainer lines, PTGMS lines, and the derived hybrids (Jiang et al. 2012; Jiang et al. 2015a; Jiang et al. 2015b; Luo et al. 2017b; Mi et al. 2018), along with high resistance to leaf and neck blast under high pressure in epidemic fields.

Molecular marker-assisted selection (MAS) is considered to be a highly efficient breeding method because it offers a rapid and precise selection of the desired genes (Tanksley et al. 1989). Application of MAS has significantly improved the rice breeding process; however, at least two disadvantages have been observed: 1) introgression of target genes had always accompanied linked chromosomal segments, thus conferring undesired traits, known as linkage drag; 2) favorable genes sometimes exhibited incomplete function due to differences in genetic backgrounds, which led to difficulties for effective and reliable trait selection. So, it is essential to perform background selection for goal-oriented breeding. Genome-wide background selection was first conceptually proposed by Xu et al. (2012). Compared with marker-assisted selection, this strategy conducted background selection using genome-wide coverage with molecular markers while making a positive selection for target genes. In previous studies, MAS had been used for single trait improvement and had already been practiced successfully for improvement in rice by whole-genome background selection. Chen et al. (2000) introduced resistance gene *Xa21*, a broad-spectrum bacterial blight (BB) resistance gene, into Minghui63, and positive selection was conducted with closely linked markers, while a series of RFLP markers, evenly distributed on the 12 chromosomes, were used to perform background selection, finally resulting in improved version ‘Minghui63^*Xa21*^’, which was the same as the original except for a fragment of less than 3.8 cM in length surrounding *Xa21*. In addition, the derived hybrid ‘Zhenshan97/Minghui63^*Xa21*^’ showed enhanced BB resistance and identical agronomic performance as that of ‘Zhenshan97/Minghui63’. High-density markers, such as SSRs and SNPs, were subsequently applied to whole-genome background selection for improving blast resistance by developing a set of NILs with seven major genes in a Basmati variety (Khanna et al. 2015). Whole-genome background selection could effectively improve complex traits, such as yield, coupled with SNP markers, and this rebuilt the genome of variety Kongyu131 by separately replacing only a small chromosome segment containing *GS3* and *Gn1a* for increased yield (Feng et al. 2017; Nan et al. 2018). For improving resistance to rice blast, MAS coupled with whole-genome background selection will be a promising approach for targeted improvement of blast resistance without significant changes in other agronomic traits. Besides, it offers a better way to develop a panel of NILs to accurately evaluate the effects of blast resistant genes in the same genetic background.

Rice breeding efficiency could also be accelerated by genomics-based technology with high-resolution SNP assays (Zhou et al. 2013). Several breeding chips developed based on high-quality re-sequencing data could lead to precise improvement by background profiling analysis (Yu et al. 2014; Chen et al. 2014). Two typical examples for employing RICE6K and RiceSNP50 in BPH resistance involved 13 genes or QTLs transferred into cultivar 9311 through positive and negative selection (Xiao et al. 2016) and wide-compatibility by stacking *f5-n* and *S5-n* loci to overcome sterility in *indica-japonica* hybrid rice (Mi et al. 2016), with reconstitution of the recurrent parent genome surpassing 99.5%. Recently, Wing et al. (2018) described a similar approach for precisely introducing a gene into the background of an elite cultivar in combination with breeding chips, designated as ‘genomic breeding’, contributing to precise incorporation of the target gene for shortening the breeding cycle and upgrading the rice varieties by genome-wide background selection. These ideals and examples confirmed that MAS with background selection in combination with breeding chips would be a promising approach for rice genetic improvement.

Two-line hybrid rice based on photoperiod- and thermo-sensitive male sterile (PTGMS) lines has greatly contributed to increase rice yields in China (Yuan 2017). Feng39S is an elite PTGMS line widely used for two-line hybrid breeding in China. It shows good characteristics with compact plant type, strong tillering ability, good rice quality, lower critical temperature point of fertility-sterility alternation, longer and more stable sterile period, and high general combining ability (Zhou et al. 2007). More than 10 combinations were developed and released using Feng39S as the female parent (www.ricedata.cn/variety/index.htm). Fengliangyou Xiang 1 (Feng39S/FXH No.1) and Fengliangyou 4 (Feng39S/9311) were two popular top two-line hybrids in China. However, most of the hybrids derived from Feng39S were highly susceptible to rice blast, which greatly limited their robust performance in rice production. In the present study, we aimed to introgress the broad-spectrum blast resistance gene *Pi2* into Feng39S through MABC. An SNP chip, RICE6K, was used for genetic background selection, especially to delete the linkage drag fragments. Our results provide a successful breeding example through MABC to carry out the precise foreground and background selection and to recover rapidly the recurrent parent genetic background to enhance resistance to blast for two-line hybrid rice.

## Results

### Introgression of blast resistance gene *Pi2* into the background of Feng39S

**Fig. 1:**
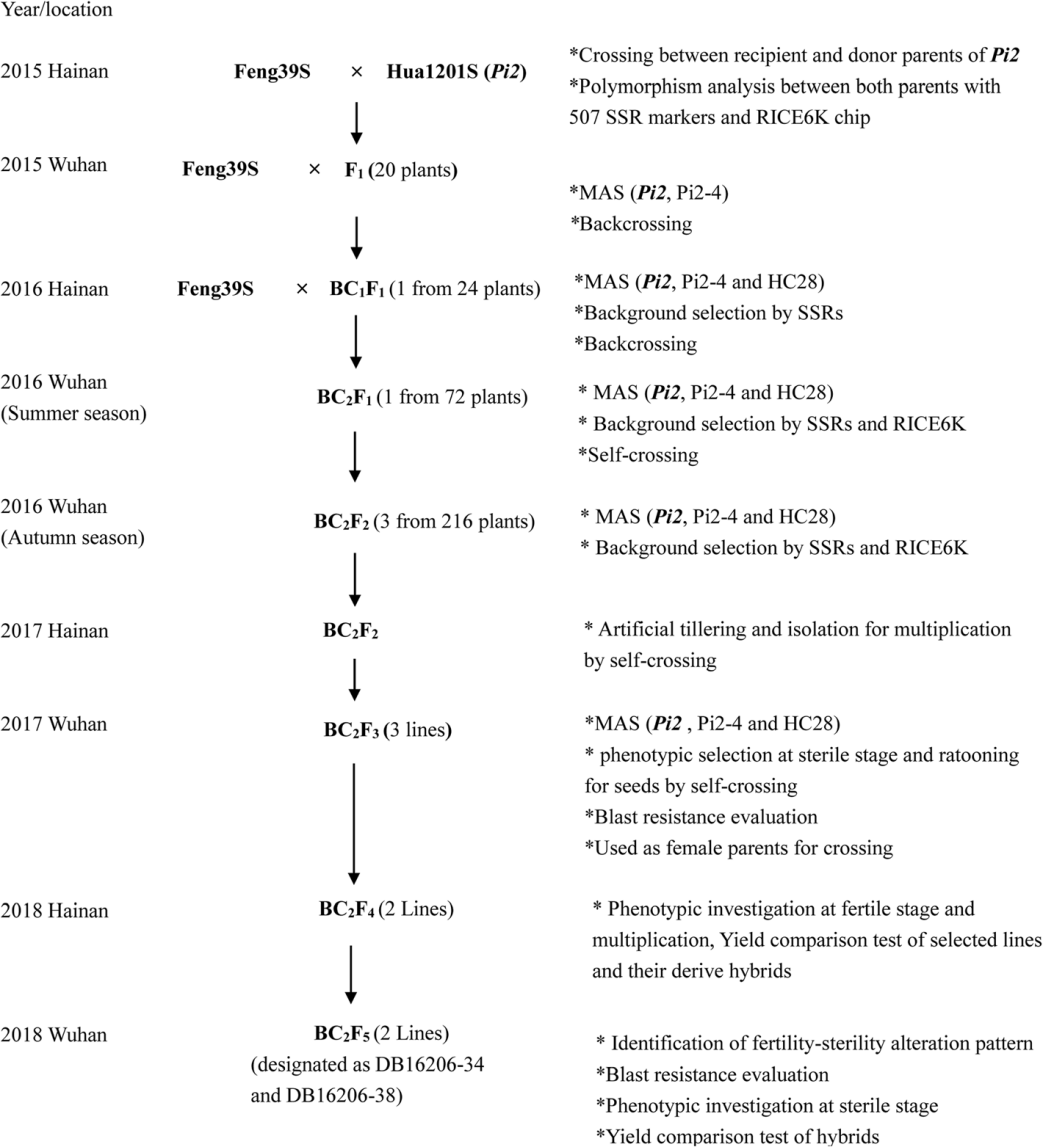
The scheme for breeding new PTGMS lines stacking *Pi2* gene by MABC

**Fig. 2:**
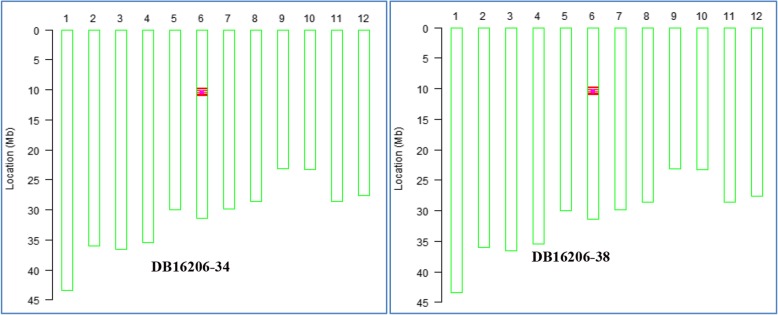
Genetic background analysis of the two selected lines of BC_2_F_2_ detected by RICE6K chip. The pink dots indicate the positions of the *Pi2* locus on chromosome 6. The red lines indicate the SNP loci with homozygous genotypes where genomic fragments of the donor parent Hua1201S were introgressed

The introgression procedure was carried out as described in Fig. 1. Twenty F_1_ plants developed by crossing between Hua1201S bearing *Pi2* (used as a donor parent of blast resistance) and the recurrent parent Feng39S under low-temperature conditions in the spring season of 2015 at Hainan were planted and tested by the tightly linked markers Pi2–4 and HC28 in the summer season in Wuhan. Eighteen individuals were confirmed positive for *Pi2*. Three F_1_ plants with the positive *Pi2* gene were transplanted in plastic pots in a greenhouse with about 20 °C of daily mean temperature for growing to a head with fertile pollens. Feng39S was backcrossed to these three fertile plants to produce BC_1_F_1_ seeds. Positive foreground selection of the *Pi2* gene by using tightly linked markers Pi2–4 and HC28 was conducted in the BC_1_F_1_ generation. A total of 155 of the 770 BC_1_F_1_ plants were identified as carrying heterozygous *Pi2*. Background selection based on 39 SSR markers of polymorphism between the donor (Hua1201S) and recurrent parent (Feng39S) was carried out for 155 *Pi2*-positive plants. One individual (the tested number was DBQ1608–22) had the highest recurrent parent genome recovery (RPGR) of 89.7%, and 35 of 39 SSR markers were restored to the background of recurrent parent Feng39S. This plant was backcrossed with Feng39S to produce BC_2_F_1_ seeds in the spring season of 2016 in Hainan. Twenty *Pi2*-positive plants were identified out of 72 BC_2_F_1_ plants in the summer season of 2016 in Wuhan. The results of PCR analysis for the remaining four SSR markers on 20 *Pi2*-positive plants showed that 12 plants were uniform with the recurrent parent. In order to select individuals with a genetic background similar to that of the recurrent parent, we analyzed the genetic background of these 12 individuals by using an SNP chip, RICE6K (Yu et al. 2014). The results showed that the similarity of genetic background among 12 selected *Pi2*-positive plants and recurrent parent Feng39S ranged from 64.83% to 92.20% (Additional file 1: Figure S1). The plant designated as DB16036–19 had the highest RPGR of 92.20%. This plant was chosen to obtain BC_2_F_2_ by self-pollination in the 20 °C greenhouse in Wuhan in the summer season of 2016. In BC_2_F_2_ of 216 plants, 36 plants carrying homozygous *Pi2* were selected by PCR of linked markers Pi2–4 and HC28 at the same time. The assayed results on the genetic background by using the RICE6K SNP chip showed that two of 36 plants, DB16206–34, and DB16206–38, were found to have the maximum genome recovery of the recurrent parent (99.67%), with only a 1.2 Mb fragment at the *Pi2* locus from the donor parent, Hua1201S (Fig. 2). The seedlings of these two selected PTGMS lines were transplanted in a field of Hainan Island in the winter-spring season of 2016–2017 for reproducing seeds by self-pollination. The harvested BC_2_F_3_ seeds were used in advanced experiments of the study.

### Blast resistance scoring in the greenhouse

The two selected PTGMS lines (DB16206–34 and DB16206–38), the recurrent parent (Feng39S), the donor parent (Hua1201S, resistant control), and CO39 (susceptible control) were scored at the seedling stage by artificial inoculation with 35 blast isolates in a greenhouse in the summer season of 2017 (Table 1). CO39 and Feng39S were highly susceptible to blast, with a susceptibility frequency of 100% and 82.86%, respectively, indicating there existed no major blast resistance gene in Feng39S. DB16206–34, DB16206–38, and Hua1201S were highly resistant against blast, with a resistance frequency of 97.14%, 97.14%, and 94.12%, respectively. Compared to CO39 (the susceptible control), we found that the recipient parent also exhibited resistance to 8 of 35 isolates in 2017. Although Feng39S was susceptible to rice blast, it may possess blast resistance genes with minor effects when infected with various isolates. So the resistance frequencies of DB16206–34 and DB16206–38 were higher than that of Hua1201S because of the resistance to an isolate (GD-17KP29) from the recurrent parent background (Table 1).

**Table 1:** Disease resistance reaction at seedling stage of breeding lines, susceptible check (CO39), and parents to 35 rice blast isolates under artificial inoculation in the greenhouse in 2017

Races of *P. grisea*	Code of isolates	CO39	Feng39S	DB16206–34	DB16206–38	Hua1201S
B01	GD-17KP01	S	S	R	R	R
B03	GD-17KP02	S	R	R	R	R
B05	GD-17KP03	S	S	R	R	R
B09	GD-17KP04	S	S	R	R	R
B13	GD-17KP05	S	S	R	R	R
B13	GD-17KP06	S	S	R	R	R
B13	GD-17KP07	S	S	R	R	R
B13	GD-17KP08	S	S	R	R	R
B13	GD-17KP09	S	R	R	R	R
B13	GD-17KP10	S	S	R	R	NA
B13	GD-17KP11	S	S	R	R	R
B13	GD-17KP12	S	S	R	R	R
B13	GD-17KP13	S	S	R	R	R
B13	GD-17KP14	S	S	R	R	R
B13	GD-17KP15	S	S	R	R	R
B15	GD-17KP16	S	R	R	R	R
B15	GD-17KP17	S	S	R	R	R
B17	GD-17KP18	S	S	R	R	R
B29	GD-17KP19	S	S	R	R	R
C05	GD-17KP20	S	S	R	R	R
C13	GD-17KP21	S	S	R	R	R
C13	GD-17KP22	S	S	R	R	R
C13	GD-17KP23	S	S	R	R	R
C13	GD-17KP24	S	S	R	R	R
C13	GD-17KP25	S	S	R	R	R
C15	GD-17KP26	S	R	R	R	R
C15	GD-17KP27	S	S	R	R	R
C15	GD-17KP28	S	S	R	R	R
C15	GD-17KP29	S	R	R	R	S
C15	GD-17KP30	S	S	S	S	S
C15	GD-17KP31	S	R	R	R	R
F01	GD-17KP32	S	S	R	R	R
F01	GD-17KP33	S	S	R	R	R
G01	GD-17KP34	S	R	R	R	R
G01	GD-17KP35	S	R	R	R	R
Number of resistant isolates		0	8	34	34	32
Number of susceptible isolates		35	27	1	1	2
Total resistance frequency (%)		0.00	17.14	97.14	97.14	94.12

Note: R: the lesion score is 1–3, indicating resistant against the blast isolate; S: the lesion score is 4–9, indicating susceptible; *NA* Not applicable

In the summer season of 2018, the selected PTGMS lines, recurrent parent Feng39S, their hybrids, two male parents (9311 and FXH No.1) for hybridizing, donor parent Hua1201S (resistant control), and CO39 (susceptible control) were inoculated at seedling stage with 22 blast isolates in a greenhouse (Table 2). The results showed that CO39 was also susceptible to all of the tested blast isolates, and Feng39S was susceptible to 19 of 22 isolates, with a susceptibility frequency of 86.36%. On the contrary, Hua1201S bearing the *Pi2* gene was resistant against 20 of 22 isolates. The two selected PTGMS lines stacking the *Pi2* gene were also resistant to 20 of 22 blast isolates, and the resistance frequency was 90.91%, the same as Hua1201S. Two male parents of the hybrids (9311 and FXH No.1) were also susceptible to blast, with susceptibility frequency of 77.27% and 90.91%, respectively. The hybrids Feng39S/9311 and Feng39S/FXH No.1, named Fengliangyou 4 and Fengliangyou Xiang 1 in farmer production, were highly susceptible to blast, with susceptibility frequency of 77.27% and 90.91%, respectively. However, the hybrids in which the selected PTGMS lines were used as female parents were highly resistant against most of the tested blast isolates, with resistance frequency of 90.91% for 9311 used as a male parent and 68.18% for FXH No.1 used as a male parent.

**Table 2:** Disease resistance reactions of breeding lines, parents, and their derived hybrids to 22 rice blast isolates under artificial inoculation in greenhouse in 2018

Races of *P.grisea*	Code of isolates	CO39	Feng39S	DB16206–34	DB16206–38	Hua1201S	DB16206–34/ 9311	DB16206–38/ 9311	Feng39S/ 9311	9311	DB16206–34/FXH No.1	DB16206–38/FXH No.1	Feng39S/FXH No.1	FXH No.1
B13	GD-18KP01	S	S	R	R	R	R	R	S	S	R	R	S	S
B13	GD-18KP02	S	S	R	R	R	R	R	S	S	R	R	S	S
B01	GD-18KP03	S	S	R	R	R	R	R	S	S	R	R	S	S
A13	GD-18KP04	S	R	R	R	R	R	R	R	S	R	R	R	S
A05	GD-18KP05	S	S	R	R	R	R	R	S	S	R	R	S	S
B13	GD-18KP06	S	S	R	R	R	R	R	S	R	R	R	S	S
C13	GD-18KP07	S	S	R	R	R	R	R	S	S	R	R	S	S
C09	GD-18KP08	S	S	R	R	R	R	R	S	R	R	R	S	S
A05	GD-18KP09	S	S	R	R	R	S	R	S	S	S	S	S	S
B13	GD-18KP10	S	S	R	R	R	R	R	S	S	R	R	S	S
B13	GD-18KP11	S	S	R	R	R	R	R	S	S	S	S	S	S
A37	GD-18KP12	S	R	R	R	R	R	R	R	R	R	R	S	S
B13	GD-18KP13	S	S	S	S	S	S	S	S	S	S	S	S	S
C05	GD-18KP14	S	S	R	R	R	R	R	S	S	S	S	S	S
C07	GD-18KP15	S	S	R	R	R	R	R	S	S	R	R	S	S
C05	GD-18KP16	S	S	R	R	R	R	R	R	R	S	R	S	S
A01	GD-18KP17	S	S	R	R	R	R	R	S	S	R	S	S	S
B13	GD-18KP18	S	S	R	R	R	R	R	S	S	R	R	S	S
C13	GD-18KP19	S	R	R	R	R	R	R	R	R	S	S	S	R
A09	GD-18KP20	S	S	S	S	S	R	R	R	R	S	S	S	S
A13	GD-18KP21	S	S	R	R	R	R	R	S	S	R	R	R	R
A13	GD-18KP22	S	S	R	R	R	R	R	S	S	R	R	S	S
Number of resistant isolates		0	3	20	20	20	20	21	5	6	15	15	2	2
Number of susceptible isolates		22	19	2	2	2	2	1	17	16	7	7	20	20
Total resistance frequency (%)		0.00	13.64	90.91	90.91	90.91	90.91	95.45	22.73	27.27	68.18	68.18	9.09	9.09

Note: R: the lesion score is 1–3, indicating resistant against the blast isolate; S: the lesion score is 4–9, indicating susceptible

### Evaluation of blast resistance in a blast epidemic field

**Fig. 3:**
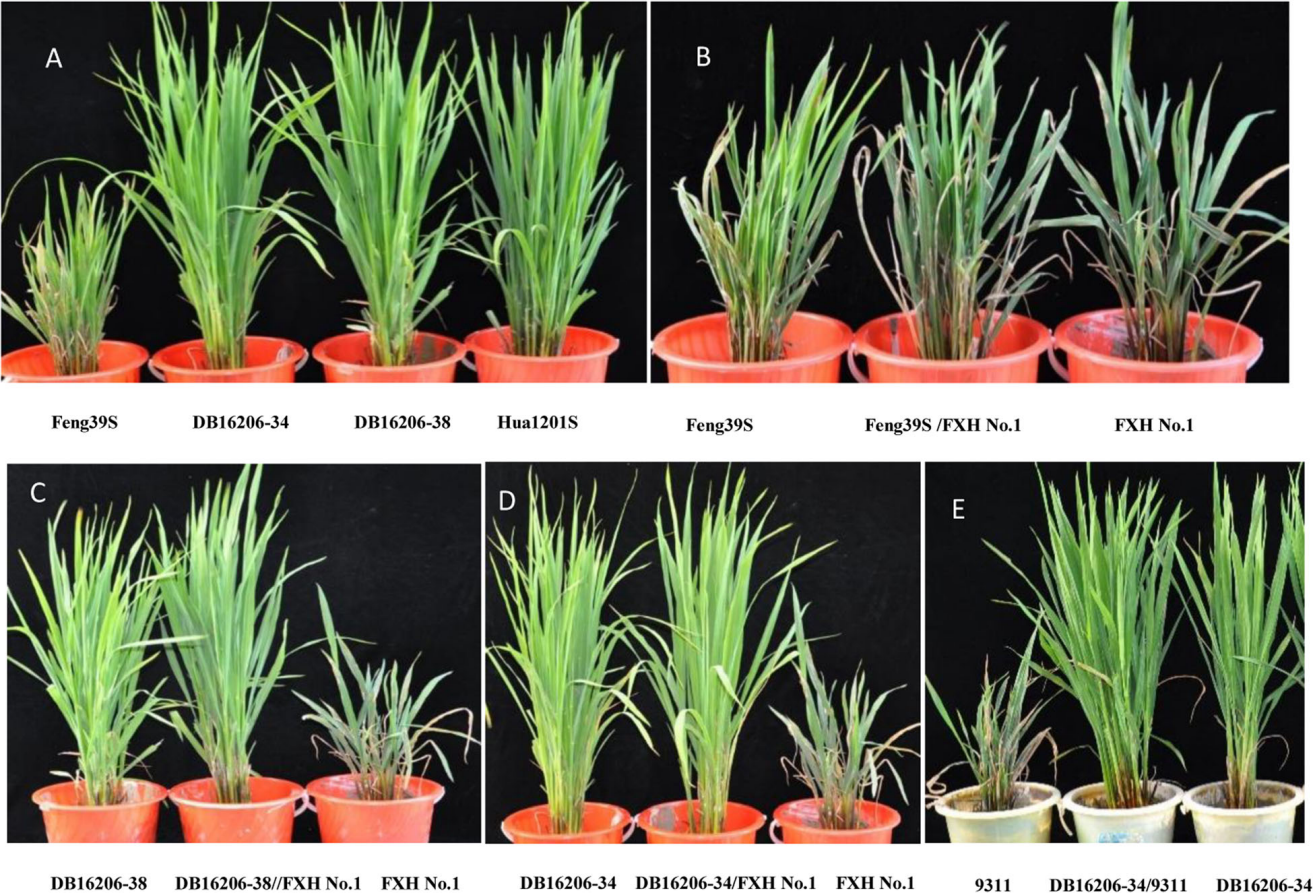
The performance for leaf blast of the two selected PTGMS lines, parents, and their hybrids in blast epidemic fields in the summer season of 2018 at Wangjia Village, Yichang City, Hubei, China. **a** the leaf blast reactions of two selected PTGMS lines and their parents. **b** the leaf blast reactions of two blast susceptible parents and the derived hybrid. **c**, **d** and **e** the leaf blast reactions of partial hybrids derived from the improved PTGMS lines crossing with male parents, FXH No.1 and 9311

Leaf and neck blast resistances are imperative for practicability in breeding blast-resistant cultivars. Therefore, we identified leaf and neck blast resistance of the two selected PTGMS lines, recurrent and donor parents, their hybrids, male parents, and the susceptible control (CO39) under two natural hotspot locations for rice blast during the summer season of 2017 and 2018, respectively (Table 3 and Fig. 3). CO39, Feng39S, Feng39S/9311, Feng39S/FXH No.1, 9311, and FXH No.1 were highly susceptible to blast at two locations, with leaf blast score ranging from 5 to 8 and neck blast infection percentage from 55.0% to 100.0%. The *Pi2* gene donor parent (Hua1201S), two selected PTGMS lines (DB16206–34 and DB16206–38) stacking the *Pi2* gene, and their hybrids (Hua1201S/9311, DB16206–34/9311, DB16206–38/9311, Hua1201S/FXH No.1, DB16206–34/FXH No.1, and DB16206–38/FXH No.1) exhibited resistance to blast in both 2 years and two locations. DB16206–34 and DB16206–38 showed resistance to blast, with a score of 2 for leaf blast and 11% for neck blast infection on average under the disease nursery in Enshi City, and with a score of 3 for leaf blast and 5.3% for neck blast infection in Yichang City in both 2017 and 2018, respectively. Also, the derived hybrids showed resistance to blast under two locations in 2018. These results suggest that enhanced and broad-spectrum resistance to blast has been obtained for DB16206–34, DB16206–38, and their hybrids, making them appropriate to replace Feng39S for developing two-line hybrid rice with blast resistance effectively.

**Table 3:** Performance for leaf and neck blast in breeding PTGMS lines, parents, and their hybrids in blast epidemic fields

Locations	Year	Entries	Leaf blast score	Neck blast infection percentage
WangjiaVillage, Yichang City, Hubei, China	2017	CO39	8	100
		Feng39S	7	66
		DB16206–34	4	10
		DB16206–38	3	7
		Hua1201S	1	4
	2018	CO39	8	79
		Feng39S	8	87
		DB16206–34	1	0
		DB16206–38	3	4
		Hua1201S	2	0
		Feng39S/9311	4	52
		Hua1201S/9311	3	8
		DB16206–34/9311	3	9
		DB16206–38/9311	3	11
		9311	5	55
		Feng39S/FXH No.1	8	100
		Hua1201S/FXH No.1	4	9
		DB16206–34/FXH No.1	4	10
		DB16206–38/FXH No.1	4	8
		FXH No.1	8	100
Lianghe Villiage, Enshi City, Hubei, China	2017	CO39	8	100
		Feng39S	8	100
		DB16206–34	2	13
		DB16206–38	2	8
		Hua1201S	2	9
	2018	CO39	8	100
		Feng39S	8	100
		DB16206–34	2	12
		DB16206–38	2	11
		Hua1201S	2	8
		Feng39S/9311	8	100
		Hua1201S/9311	2	13
		DB16206–34/9311	2	13
		DB16206–38/9311	3	9
		9311	7	100
		Feng39S/FXH No.1	8	100
		Hua1201S/FXH No.1	4	20
		DB16206–34/FXH No.1	4	21
		DB16206–38/FXH No.1	4	20
		FXH No.1	8	100

### Characterization of fertility-sterility alternation in plant growth chambers and under natural field conditions

The two selected PTGMS lines (DB16206–34 and DB16206–38), recurrent parent Feng39S, and donor parent Hua1201S were treated in plant growth chambers in which the daily mean temperature (DMT) was set for 21 °C, 22 °C, 23 °C, 24 °C, and 25 °C with identical day length as 14 h for 12 consecutive days (Jiang et al. 2015a). The investigated results for pollen fertility-sterility alternation behavior showed that DB16206–34 and DB16206–38 were completely male sterile, with more than 99.5% pollen sterility when the DMT exceeded 22 °C. Partial fertility was observed under 21 °C, the pollen fertility-sterility alternation behavior was the same as that of recurrent parent Feng39S (Table 4), and their critical temperature point (CTP) may be located at 22 °C of DMT. However, the CPT of Hua1201S may be located at 24 °C, slightly higher than that of Feng39S.

**Table 4:** Fertility-sterility alternation behavior of two selected PTGMS breeding lines and parents under five temperature regimes in growth chambers with 14-h light duration

Entries	Pollen sterility (%)				
	21 °C	22 °C	23 °C	24 °C	25 °C
Hua1201S	64.76	86.06	98.38	99.47	100.00
Feng39S	88.35	99.84	99.88	99.99	100.00
DB16206–34	88.33	99.51	100.00	100.00	100.00
DB16206–38	74.85	99.59	99.93	100.00	100.00

**Fig. 4:**
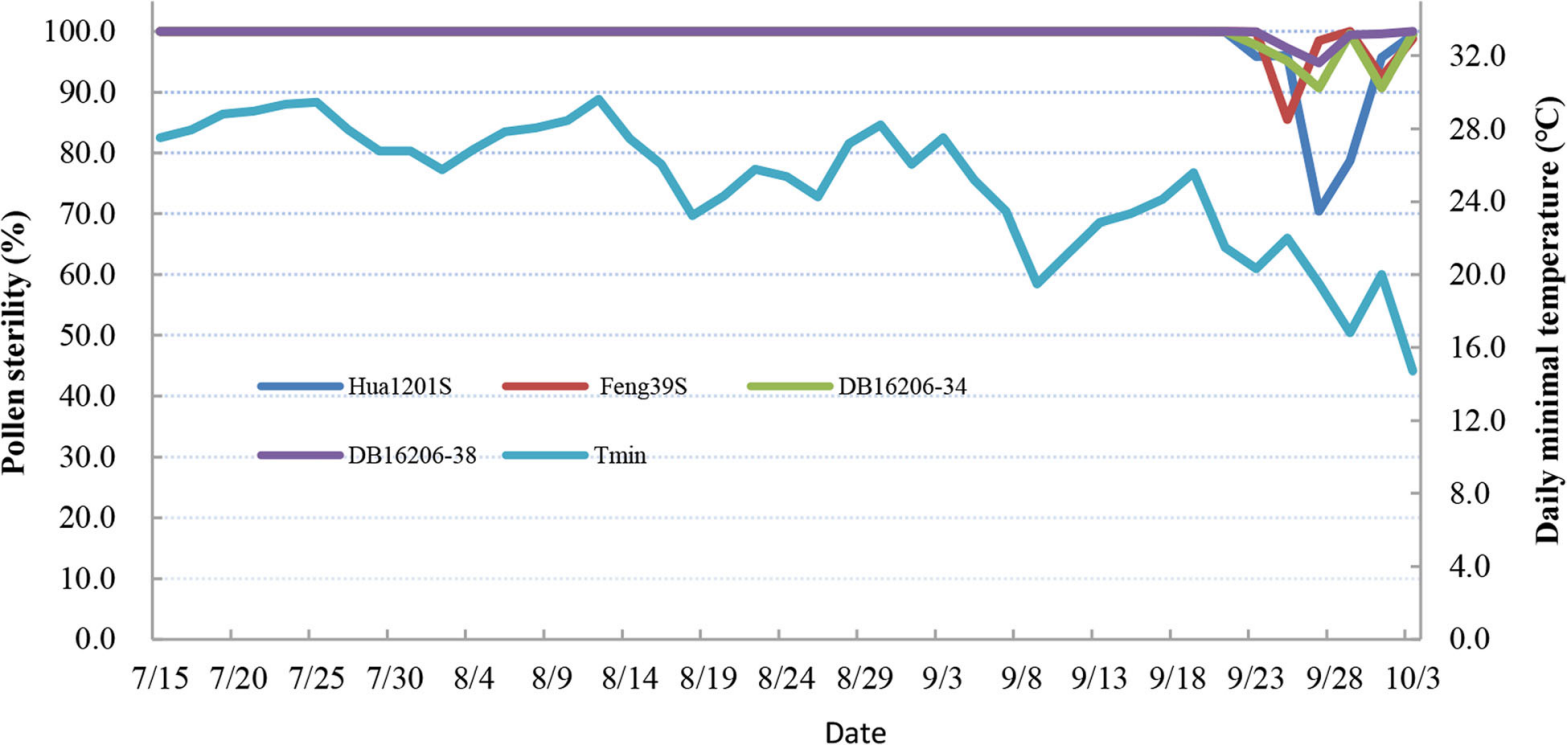
Pollen fertility dynamic performance of selected breeding PTGMS lines and parents in the summer season of 2018 in Wuhan

We further investigated the stable sterile period of DB16206–34 and DB16206–38, Feng39S, and Hua1201S by observing dynamic pollen fertility expression patterns in the experiment farm field of HAU in the summer season of 2018. The results indicated that DB16206–38 was completely male sterile (pollen sterility surpassing 99.5%) from 15 July to 23 September, possessing a stable sterile period of 70 days, which is identical to that of Feng39S. The stable sterility duration of DB16206–34 was 68 days from 15 July to 21 September, which was identical to that of Hua1201S. When the temperature decreased, and daylength in early September became shorter, their fertilities alternated from completely sterile to partially fertile in late September (Fig. 4).

### Agro-morphological characters and rice quality of the selected PTGMS lines and recurrent parent

The two selected PTGMS lines and recurrent parent Feng39S were planted in the fertile phase in the winter season of 2017–2018 in Hainan three times, on 20 and 30 November and 10 December 2017. Seven agronomic traits (days to heading, plant height, panicle number, panicle length, spikelets per panicle, filled-grain percentage, and 1000-grain weight) were recorded. Ten rice quality traits, brown rice percentage (%), milled rice percentage (%), head rice percentage (%), chalky rice percentage(%), chalkiness degree(%), rice grain length (mm), grain length/width ratio, alkali spreading value, amylose content(%), and gel consistency(mm), were analyzed. The lines were planted in the sterile phase in the summer season of 2018 in Wuhan seven times, from 1 April to 1 July 2018, with an interval of 15 days. Six agronomic traits (days to heading, plant height, panicle number, panicle length, spikelets per panicle, and stigma exsertion rate) were recorded. The average data of agro-morphological characters showed that most of the agronomic traits and rice quality among the selected PTGMS lines and recurrent parent had no differences except for the plant height of DB16206–34 in the sterile phase in Wuhan, which was higher than that of Feng39S by 1.8 cm, and rice grain length/width ratio in the fertile phase in Hainan was lower than that of Feng39S by 0.2, at 5% significance by the *T*-test, respectively (Tables 5, 6 and Additional file 2: Figure S2).These results indicated that the agro-morphological characters of the two selected PTGMS lines by MABC reverted to the characters of the recurrent parent.

**Table 5:** Agronomic performance of selected PTGMS lines and recurrent parent Feng39S

Phase, season, and sites	Entries	DTH^a^	PH	PN	PL	SPP	FGP	GW	SER
Sterile phase in summer season of 2018 in Wuhan	Feng39S	84.6 ± 11.7	87.7 ± 2.0	9.0 ± 1.0	23.8 ± 1.2	155.7 ± 22.7	–	–	22.5 ± 10.4
	DB16206–34	84.9 ± 11.6	89.5 ± 2.4^b^	9.1 ± 1.0	23.9 ± 1.0	159.0 ± 17.7	–	–	22.6 ± 7.8
	DB16206–38	84.4 ± 11.4	88.5 ± 2.4	9.5 ± 1.0	23.9 ± 1.0	165.9 ± 18.7			30.08 ± 10.1
Fertile phase in winter season of 2017–2018 in Hainan	Feng39S	105.3 ± 1.2	72.2 ± 0.2	9.1 ± 1.1	20.0 ± 0.3	149.5 ± 14.9	29.3 ± 10.9	22.9 ± 1.7	–
	DB16206–34	105.3 ± 2.1	73.3 ± 1.2	8.4 ± 1.2	20.6 ± 0.2	165.4 ± 4.2	25.6 ± 3.9	22.3 ± 1.1	–
	DB16206–38	105.3 ± 2.1	73.5 ± 1.0	8.6 ± 1.0	20.2 ± 0.8	142.0 ± 8.6	38.6 ± 12.4	23.0 ± 0.5	–

^a^*DTH* Days to heading (d), *PH* Plant height (cm), *PN* Panicle number per plant, *PL* Panicle length (cm), *SPP* Spikelets per panicle, *FGP* Filled-grain percentage (%), *GW* 1000-grain weight (g), *SER* Stigma exsertion percentage(%)

^b^Symbols following after means indicate significant at the 5% significance level by the *T*-test

**Table 6:** The rice quality performance of selected PTGMS lines and recurrent parent Feng39S

Entries	BRP^a^	MRP	HRP	CRP	CD	RGL	L/W	ASV	AC	GC
Feng39S	79.6 ± 0.1	69.9 ± 0.3	64.2 ± 0.4	0.6 ± 0.2	0.2 ± 0.2	6.0 ± 0.1	3.0 ± 0.0	6.9 ± 0.1	12.9 ± 0.7	79.6 ± 1.8
DB16206–34	79.7 ± 0.1	70.0 ± 0.2	64.1 ± 0.4	0.1 ± 0.1	0.0 ± 0.0	6.0 ± 0.1	2.9 ± 0.0	7.0 ± 0.0	12.8 ± 0.2	80.5 ± 3.2
DB16206–38	78.7 ± 0.1^b^	69.5 ± 0.2	64.0 ± 0.2	0.6 ± 0.4	0.1 ± 0.1	5.9 ± 0.0	2.8 ± 0.0*	6.9 ± 0.1	12.4 ± 0.7	82.6 ± 2.8

^a^*BRP* Brown rice percentage (%), *MRP* Milled rice percentage (%), *HRP* Head rice percentage (%), *CRP* Chalky rice percentage (%), *CD* Chalkiness degree (%), *RGL* Rice grain length (mm), *L/W* Grain length/width ratio, *ASV* Alkali spreading value, *AC* Amylose content (%), *GC* Gel consistency (mm)

^b^Symbols following after means indicate significant at the 5% significance level by the *T*-test

### Yield, agronomic traits, and rice quality performance of hybrids in multi-site trials

The two selected PTGMS lines (DB16206–34 and DB16206–38) and recurrent parent Feng39S were used as female parents to produce hybrid F_1_ seeds with two male parents (9311 and FXH No.1) in the summer season (sterile phase) of 2017. Feng39S/9311 and Feng39S/FXH No.1 were named Fengliangyou 4 and Fengliangyou Xiang 1 by the China National Crop Variety Approval Committee and planted extensively as one-crop types in the rice-growing area of the middle and lower reaches of the Yangtze River. DB16206–34/9311, DB16206–34/FXH No.1, DB16206–38/9311, and DB16206–38/FXH No.1 were tested at six sites using the method described by the China National Crop Variety Approval Committee, with Feng39S/9311 and Feng39S/FXH No.1 used as controls in 2018 (Table 7). The results showed that the average yields of DB16206–34/9311 and DB16206–38/9311 were 10.3 and 10.2 t/ha, respectively, slightly higher than the yield (9.9 t/ha) of the control combination (Feng39S/9311), although statistically insignificant. DB16206–34/FXH No.1 and DB16206–38/FXH No.1 yielded 10.1 and 10.2 t/ha, respectively, almost the same as the yield of Feng39S/FXH No.1. We investigated five agronomic traits (days to maturity, plant height, spikelets per panicle, filled-grain percentage, and 1000-grain weight) and recorded the average data of six locations (Table 7). Non-significant differences were found between the new combinations and corresponding controls. The rice quality of grains from six test sites was analyzed. Eight rice quality characters (brown rice percentage, head rice percentage, chalky rice percentage, rice grain length, grain length/width ratio, alkali spreading value, amylose content, and gel consistency) showed statistically non-significant differences existing among the new combinations and controls (Table 7). These results showed that the new combinations with blast resistance had the same yield, agronomic traits, and rice quality as the control hybrids with blast susceptibility and strongly verified that the genetic background of the selected PTGMS lines reverted to that of the recurrent parent except for an introgressed fragment of the *Pi2* locus on chromosome 6.

**Table 7:** The performance of yield, main agronomic traits, and rice quality of hybrids in multi-site trials

Entries	Sites	YD	DTM	PH	SPP	FGP	GW	BRP	HRP	CRP	RGL	L/W	ASV	AC	GC
DB16206–34/9311	Wuhan	9.9	124.0	130.1	173.1	80.6	29.1	78.7	65.8	13.7	6.2	3.0	6.8	16.1	94.1
DB16206–34/9311	Ezhou	8.8	126.0	135.4	210.1	77.7	25.3	79.6	65.3	12.2	6.2	3.0	6.8	14.9	89.6
DB16206–34/9311	Xiaogan	11.0	121.0	135.8	215.7	88.9	27.4	78.8	66.2	29.2	6.1	3.0	5.6	14.7	84.2
DB16206–34/9311	Xiangyang	10.1	139.0	136.4	210.8	84.6	27.4	78.9	65.6	18.7	6.1	2.9	5.8	15.0	87.0
DB16206–34/9311	Jingzhou	11.9	137.0	126.0	157.3	80.1	27.7	78.9	63.0	5.9	6.4	3.1	6.4	17.2	90.0
DB16206–34/9311	Hainan	10.3	130.0	96.3	161.8	79.9	28.5	79.1	67.7	5.0	6.1	2.7	6.8	16.8	89.8
Average		10.3	129.5	126.7	188.1	81.9	27.6	79.0	65.6	14.1	6.2	3.0	6.4	15.8	89.1
DB16206–38/9311	Wuhan	9.6	124.0	131.0	167.8	80.3	29.1	78.6	65.3	10.6	6.2	3.0	6.6	17.0	95.8
DB16206–38/9311	Ezhou	8.9	126.0	135.4	196.9	73.0	26.2	79.2	53.6	19.7	6.0	3.0	6.4	14.9	86.9
DB16206–38/9311	Xiaogan	10.9	121.0	134.6	237.3	91.7	28.0	79.0	65.8	30.0	6.2	3.0	5.8	13.9	85.3
DB16206–38/9311	Xiangyang	9.4	139.0	134.6	211.6	81.9	26.7	78.6	66.5	17.4	6.2	3.0	5.9	16.0	87.4
DB16206–38/9311	Jingzhou	11.8	135.0	126.0	154.3	89.3	27.2	79.6	62.3	5.4	6.4	3.1	6.2	16.5	87.3
DB16206–38/9311	Hainan	10.5	130.0	96.9	167.5	79.7	27.4	79.0	68.3	4.5	6.2	2.7	6.9	16.4	88.6
Average		10.2	129.2	126.4	189.2	82.6	27.4	79.0	63.6	14.6	6.2	3.0	6.3	15.8	88.5
Feng39S/9311	Wuhan	9.5	124.0	129.6	166.9	80.3	29.1	79.6	66.1	10.2	6.3	3.0	6.7	17.0	94.8
Feng39S/9311	Ezhou	8.6	126.0	137.5	206.6	73.1	26.5	79.9	64.7	17.3	6.2	3.0	6.7	14.7	90.7
Feng39S/9311	Xiaogan	10.2	121.0	132.6	213.7	92.1	28.0	79.3	66.1	35.3	6.2	3.0	5.8	14.0	84.5
Feng39S/9311	Xiangyang	9.3	139.0	134.0	186.6	83.0	27.5	79.2	65.9	17.3	6.2	3.0	5.9	15.4	89.5
Feng39S/9311	Jingzhou	11.1	137.0	129.0	189.7	93.5	28.8	79.8	63.5	5.8	6.6	3.1	6.4	18.0	91.3
Feng39S/9311	Hainan	10.7	130.0	95.6	176.5	77.6	27.6	78.8	67.5	4.7	6.2	2.7	6.9	16.8	90.3
Average		9.9	129.5	126.4	190.0	83.3	27.9	79.4	65.6	15.1	6.3	3.0	6.4	16.0	90.2
DB16206–34/FXH No.1	Wuhan	9.7	123.0	130.8	174.0	83.8	26.9	79.6	68.6	9.0	6.2	2.9	6.7	13.6	95.7
DB16206–34/FXH No.1	Ezhou	9.2	124.0	133.6	182.1	82.5	25.8	80.0	62.4	11.9	6.0	3.0	6.6	13.7	86.0
DB16206–34/FXH No.1	Xiaogan	9.7	117.0	126.8	199.4	92.4	26.9	79.4	67.8	21.5	6.2	2.9	5.7	13.6	88.4
DB16206–34/FXH No.1	Xiangyang	9.6	135.0	127.4	224.4	91.1	27.9	79.7	68.0	4.5	6.2	3.0	5.1	14.2	87.6
DB16206–34/FXH No.1	Jingzhou	11.4	137.0	127.0	158.3	92.0	27.2	80.1	64.4	4.9	6.4	3.1	6.4	15.9	81.3
DB16206–34/FXH No.1	Hainan	11.1	126.0	93.4	147.7	87.6	25.9	80.0	70.1	1.7	6.1	2.8	6.7	15.2	88.4
Average		10.1	127.0	123.2	181.0	88.2	26.8	79.8	66.9	8.9	6.2	3.0	6.2	14.4	87.9
DB16206–38/FXH No.1	Wuhan	9.3	123.0	129.9	154.3	85.4	26.6	79.7	68.6	8.5	6.1	3.0	6.5	14.0	94.3
DB16206–38/FXH No.1	Ezhou	9.0	124.0	131.9	179.9	83.2	25.2	80.0	61.3	11.7	6.1	3.0	6.0	13.4	87.5
DB16206–38/FXH No.1	Xiaogan	10.6	120.0	130.8	222.2	90.9	27.0	79.5	68.9	15.3	6.2	2.9	5.6	13.8	89.8
DB16206–38/FXH No.1	Xiangyang	9.5	135.0	126.0	197.8	94.9	27.7	79.5	66.8	6.0	6.1	2.9	5.2	14.6	89.9
DB16206–38/FXH No.1	Jingzhou	11.8	136.0	131.0	147.7	91.4	26.5	79.9	64.5	3.1	6.4	3.1	6.2	15.7	80.6
DB16206–38/FXH No.1	Hainan	10.8	128.0	93.6	172.1	83.6	25.2	79.3	69.3	2.6	6.1	2.8	6.8	15.1	91.7
Average		10.2	127.7	123.9	179.0	88.2	26.4	79.7	66.6	7.9	6.2	3.0	6.1	14.4	89.0
Feng39S/FXH No.1	Wuhan	9.6	123.0	129.9	190.9	75.0	25.9	79.6	68.5	7.8	6.2	3.0	6.6	14.7	96.6
Feng39S/FXH No.1	Ezhou	8.9	124.0	133.4	207.4	81.6	25.4	80.2	62.4	11.0	6.1	3.0	6.1	13.8	89.6
Feng39S/FXH No.1	Xiaogan	10.2	120.0	134.4	189.2	92.0	27.2	79.7	68.4	25.0	6.2	2.9	5.7	13.8	88.9
Feng39S/FXH No.1	Xiangyang	10.1	135.0	127.2	229.3	94.0	26.8	80.8	68.8	4.1	6.2	2.9	5.2	14.2	87.5
Feng39S/FXH No.1	Jingzhou	11.6	136.0	130.0	175.3	92.8	26.6	81.1	65.4	5.0	6.4	3.1	6.4	15.3	83.5
Feng39S/FXH No.1	Hainan	10.5	126.0	91.8	166.6	92.3	27.3	80.0	69.9	2.5	6.1	2.8	6.9	15.2	90.4
Average		10.1	127.3	124.5	193.1	87.9	26.5	80.2	67.2	9.2	6.2	3.0	6.2	14.5	89.4

*YD* Yield (t/ha), *DTM* Days to maturity, PH: Plant height (cm), *SPP* Spikelets per panicle, *FGP* Filled-grain percentage (%), *GW* 1000-grain weight (g), *BRP* Brown rice percentage (%), *HRP* Head rice percentage (%), *CRP* Chalky rice percentage (%), *RGL* Rice grain length (mm), *L/W* Grain length/width ratio, *ASV* Alkali spreading value, *AC* Amylose content (%), *GC* Gel consistency (mm)

## Discussion

The two-line hybrid rice breeding system based on PTGMS lines has some advantages over three-line hybrid types, such as the wide choice of restorer lines for improving the probability of selecting combinations with high yield feasible for *indica-japonica* hybrids, with no negative cytoplasmic effects, and simplified hybrid seed production procedures with one line of dual-use (Yang et al. 2007). However, two-line hybrid rice production is severely limited because of various diseases, of which blast is considered as the most destructive, leading to large yield losses (Ashkani et al. 2016). In the present study, we effectively introduced blast resistance gene *Pi2* into the elite PTGMS line Feng39S through MABC with precise foreground selection of tightly linked molecular markers and genetic background selection with an SNP breeding chip in each generation. We selected two plants with recurrent parent genome recovery of 99.67% in the BC_2_F_3_ generation; these breeding examples had one of the highest RPGRs, which helped to shorten the breeding cycle up to 2–3 years. Except for the resistance to rice blast being significantly improved, the agronomic characters, yield, and rice quality of selected PTGMS lines and derived hybrid combinations were highly consistent with those of the recurrent parent and its hybrids, which fully proved that the results of this study demonstrated a very successful MABC breeding case.

The purpose of MABC breeding is to achieve an effective reduction in the donor genome proportion to retain the characteristics of the recurrent parent in improved varieties apart from the introgression of one or more target genes for favorable traits (Hasan et al. 2015). For conventional backcrossing breeding, at least six times backcrossing was combined with phenotypic selection to achieve the expected RPGR of 99.2% theoretically (Acquaah 2007). However, background selection could extremely accelerate RP recovery by BC_4_, BC_3,_ or even BC_2_, thus saving two to four generations for ‘complete line replacement’ (Ribaut et al. 2002). MABC is a developed efficient method by which using relatively large population sizes for the BC_1_ generation, it is possible to recover the recurrent parent genotype using only two or three backcrosses. In our study, parental polymorphism SSR markers were applied for background selection in BC_1_, and one individual both carrying *Pi2* and possessing the highest RPGR was further backcrossed with Feng39S to generate BC_2_. The remaining SSR markers and additional developed markers based on RICE6K chip analysis between Hua1201S and Feng39S were integrated into background selection after positive selection in BC_2_F_1_. Some individuals with the least number of polymorphic markers were then examined by RICE6K, and the results showed that one plant, designated as DB16036–19, possessed RPGR of 92.20%. In BC_2_F_2_, both foreground selection and background selection were performed to eliminate residual fragments of donors and to obtain individuals with homozygous *Pi2*. RICE6K chip analysis for selected plants revealed that two plants, designated as DB16206–34 and DB16206–38, harbored homozygous resistance gene *Pi2* and had identical genetic background as recurrent parent Feng39S. There is little reliance on complex phenotypic selection and breeder’s experience. Therefore, we recommend that MABC strategy be coupled with background selection. This would contribute to shortening the breeding cycle and largely reducing dependence on stringent phenotypic selection, especially using high-resolution breeding chips for background selection.

In conventional MAS breeding procedures, background selection were conducted just combing with hundreds of RFLP or SSR markers for screening polymorphism between different parents, which was low-efficiency and low-coverage (Chen et al. 2000; Ahmed et al. 2016). In the present study, background selection was performed by using 507 SSR markers, of which only 39 were polymorphic between parents, showing the closed genetic relationship between parents and inefficient at high costs. However, the sequence data of rice accessions are rapidly increasing, which offers abundant and evenly distributed SNPs for integrating into SNP arrays with high resolution (Zhou et al. 2013). Owing to SNP array detection, we could develop more markers purposeful and obtain individuals with higher genome recovery. SNP arrays could also be applied for genetic background recovery examination of the developed lines by MAS (Jiang et al., 2015a, b; Wang et al. 2016), then employed to develop a panel of NILs for evaluation gene effects and pyramiding breeding (Xiao et al. 2016; Mi et al. 2016). Besides, the SNP array could be applied for gene diagnosis and further targeted improvement for existing breeding lines in MAS, for example, Zhou et al. (2018) reported that gene diagnosis for a blast resistance line KP2, carrying *Pi1* and *Pi2*, which was with delayed heading date compared with the recipient parent KY131. RICE60K (another high-resolution SNP array) analysis showed that *Hd1* closely linked to *Pi2*, thereafter, recombination selection was performed for breaking the linkage drag of *Hd1* using MAS.

The neck blast is considered as the most destructive form of rice blast disease, and improvement and deployment of rice varieties with a high level of resistance to neck blast is the most effective approach to control blast disease in rice production. Several previous reports have proved *Pi2* gene conferred resistance to neck blast (Jiang et al. 2012; Jiang et al., 2015a, b; Mi et al. 2018). The results of blast evaluation conferred that *Pi2* was a broad-spectrum and stable blast R gene in present study. The donor parent Hua1201S bearing the *Pi2* gene was resistant against 52 of 56 tested *M. oryzae* isolates in the greenhouse, and the scores of leaf blast were 1 or 2, and the neck blast infection percentages were lower than 10% in two blast epidemic areas for 2 years. The two selected PTGMS lines stacking the *Pi2* gene from Hua1201S had similar performance for blast resistance. However, the blast resistance under the *Pi2* gene heterozygous in hybrids expressed some different phenotypes. In the greenhouse, the resistance frequencies of DB16206–34/9311 and DB16206–38/9311 were higher than 90%, but those of DB16206–34/FXH No.1 and DB16206–38/FXH No.1 were lower than 70%. Under natural blast epidemic field conditions, the resistance to leaf and neck blast of DB16206–34/9311 and DB16206–38/9311 was also stronger than that of DB16206–34/FXH No.1 and DB16206–38/FXH No.1, indicating that the resistance of the male parent would affect the performance of hybrids while the same PTGMS lines harboring the blast R gene *Pi2* were used as female parents. The popular *indica* cultivar 9311 is a core restorer line extensively employed in hybrid rice breeding procedures in China, because of its high general combining and good level of blast resistance (Dai et al. 1997). Yang et al. (2009) firstly mapped a blast resistance gene *Pi41*, and another two novel blast resistance genes, *Pi60(t)* and *Pi61(t)*, were identified and mapped, showing resistance frequency as high as 86.5% of 495 strains collected from *indica* and *japonica* derived rice materials (Lei et al. 2013), and *Pi41* conditioned complementary reactions to another 15 blast R genes (including *Pi2*) under infection with blast isolates (Yang et al. 2009), thus represents a enhanced component for R gene-stacking for enhanced blast resistance in hybrid rice. So our results implied that both parental lines would be beneficial to enhance and stabilize blast resistance in rice breeding. Overall, compared with the blast resistance of recurrent parent Feng39S and its derived hybrids, the blast resistance of the selected PTGMS lines and their derived hybrids increased significantly (Tables 1, 2 and 3). DB16206–34/9311 and DB16206–38/9311 as blast-resistant versions could replace Feng39S/9311 (Fengliangyou 4) to plant in farmers’ fields. DB16206–34/FXH No.1 and DB16206–38/FXH No.1 could also replace Feng39S/FXH No.1 (Fengliangyou Xiang 1) to plant in farmers’ fields.

The critical temperature point (CTP) of fertility-sterility alternation of PTGMS lines refers to the change in pollen from fertile to sterile phase or vice verse at a given temperature point, and stable sterility duration (SSD) refers to the duration of time (days) keeping the pollen completely sterile at a given site (Virmani et al. 2003). These two parameters are important for the safety of two-line hybrid seed production at a given site. Feng39S has a relatively lower CPT (22 °C of daily mean temperature) and longer SSD (> 70 days), which may be one of the reasons why it is widely used in China. The two selected PTGMS lines (DB16206–34 and DB16206–38) bearing the *Pi2* gene in the study have similar CTP and SSD as Feng39S (Table 4 and Fig. 4). This fully demonstrates the superiority of genome-wide background selection in MABC breeding programs. Previous studies revealed that two major dominant genes and minor polygenes were conditioning the CTP (He et al. 1999; Tao et al. 2003). MAS for CTP could not be adopted because the mechanism of CTP was still poorly understood (Jiang et al. 2015a; Jiang et al. 2015b; Mi et al. 2018). In our study, we also proposed a new strategy for developing PTGMS line by using another low CTP line as donor, contributing to breeding stable CTP lines without MAS because of the male-sterile of PTGMS trait were controlled by recessive genes, including the major or minor loci. Furthermore, because of no segregation of sterile trait, we need not perform genotypic selection for major loci such as *pms3* and *tms5* (Ding et al. 2012; Zhou et al. 2014), or phenotypic selection for pollen sterility at heading stage during almost all selective generations.

The characterization of the selected PTGMS lines and their derived hybrids showed that their yield, growth duration, main agronomic traits, and rice quality were almost the same as those of the recurrent parent and its derived hybrids (Tables 5, 6, and 7). In the trials of hybrids of six sites, the average data for each character were not significantly different by the *T*-test. Feng39S/9311, which was named Fengliangyou 4 in farmer production, has been one of the most popular two-line hybrids as one cropping type in the rice-growing areas of the middle and lower reaches of the Yangtze River, the largest rice-growing area in China in the past 10 years. DB16206–34/9311 or DB16206–38/9311 can be blast-resistant versions to replace Fengliangyou 4 for planting in farmer production. Feng39S/FXH No.1, named Fengliangyou Xiang 1, has been one of the most popular two-line hybrids as ratoon rice in the past 10 years in Hubei Province, China. DB16206–34/FXH No.1or DB16206–38/FXH No.1 can also be used as blast-resistant versions to replace Fengliangyou Xiang 1 for release. Our evaluation is the first successful case to apply MABC with genomics-based background selection to improve the blast resistance of PTGMS lines for two-line hybrid rice breeding.

## Materials and methods

### Plant materials and the selection process

Hua1201S, which was developed from the offspring of Guangzhan63-4S^4^/VE6219, was an *indica* PTGMS line carrying *Pi2* conferring broad-spectrum and high resistance to rice blast, and was used as a *Pi2* gene donor parent (Mou et al. 2017). VE6219 was derived from ‘T1007^4^/C101A51’ by MABC. ‘C101A51’ was a rice germplasm resource carrying *Pi2* gene (Mackill and Bonman 1992). Our group developed several PTGMS lines against blast in past years, such as Hua1037S and Hua1228S (Jiang et al. 2015; Mi et al. 2018) through taking VE6219 as *Pi2* donor. Feng39S was developed from Guangzhan63S through ion beam radiation by Hefei Fengle Seed Co., Ltd. (Zhou et al. 2012), and Guangzhan63S, Guangzhan63-4S, Feng39S and Hua1201S shares relatively similar genetic background in terms of pedigree resource, and differences in genomic proportions between Hua1201S and Feng39S has been shown in Additional file 3: Figure S3 Several two-line hybrid rice varieties were derived, released, and planted in a large area by using Feng39S as the female parent with high yield potential, combining ability, and rice quality, but susceptibility to blast. Feng39S was used as a recurrent parent (RP) in the study for improving its blast resistance by stacking the blast R gene *Pi2* from Hua1201S through marker-assisted backcrossing. A cross was made between Hua1201S and Feng39S, and then two successive backcrossings and four generations of self-pollination were performed (Fig. 1). Foreground selection was conducted with tightly linked flanking InDel marker Pi2–4 and SSR marker HC28 for the presence of the *Pi2* locus in each generation, and background selection was performed from BC_1_F_1_ to BC_2_F_2_ by using SSR markers and the RICE6K breeding chip (Yu et al. 2014). During background selection, the individual plants, possessing the highest genome recovery of recurrent parent, were selected according to the percentage of the number of markers consistent with the recipient parent (Feng39S) to the total number of detected markers, and then tested for RICE6K analysis.

### DNA extraction, molecular markers, and PCR amplification

Total genomic DNA was extracted from fresh rice leaves according to the protocol described by Dellaporta et al. (1983). Two flanking molecular markers, Pi2–4 and HC28, were used to confirm the presence of the *Pi2* locus for positive foreground selection. Pi2–4 (F:5′-CGGTAAGAGTAACACCAAGC -3′, R:5′-GACGTGCGAGTTGTGACAGCT-3′) was located upstream of the *Pi2* gene by 23 kb, and HC28 (F:5′-TCCAAGACTGAACAGCGAGA-3′, R:5′-TGCGAATCAAATCACTGCAC-3′) was located downstream of the *Pi2* gene by 45 kb (Jiang et al. 2012). PCRs were conducted as stated previously by Mi et al. (2016). PCRs were performed on a MyCycler™ thermal cycler(Bio-Rad, USA), with 20 μl reaction mixture containing 2 μl of genomic DNA (10 ng/μl), 2 μl of 10 × buffer,0.2 μl of each primer (10 μM), 1.4 μl of MgCl_2_ (25 mM), 2 μl of dNTP (2 mM), 0.2 μl of Taq polymerase (5 U/μl), and 12 μl of H_2_O.The PCR amplification procedure consisted of one cycle of pre-denaturation at 94 °C for 5 min, followed by 35 cycles of denaturation at 94 °C for 30 s, annealing at 55 °C for 30 s, and extension at 72 °C for 40 s, and then a final extension at 72 °C for 5 min. The amplified products were then electrophoretically resolved on a 4% denaturing polyacrylamide gel in 0.5 × TBE buffer.

### Rice blast resistance evaluation

The screening of seedling leaf blast was conducted in the Plant Protection Institute of Guangdong Academy of Agricultural Sciences, China. The two selected PTGMS lines, recurrent parent, donor parents, and susceptible control CO39 were inoculated at seedling stage in the greenhouse with 35 isolates of *M. oryzae* in 2017. The two selected PTGMS lines, recurrent parent and their derived hybrids, two male parents, donor parent, and susceptible control CO39 were inoculated at seedling stage in the greenhouse with 22 isolates of *M. oryzae* in 2018. The resistance frequency (%) was calculated as: the number of resistant (non-infected) strains divided by the total number of inoculated strains. Leaf and neck blast resistance were identified in 2017 and 2018 under natural conditions by planting in the rice blast disease epidemic fields of two locations, Wangjia Village of Yuan-An County and Lianghe Village of Enshi City, Hubei Province, where rice blast disease was epidemic every year. These two disease nurseries had been the standard nursery of rice blast evaluation in the China national and Hubei provincial regional trials of rice varieties for more than 30 years. The standard evaluation system for rice (SES) (IRRI 2002) was used to assess the disease resistance reaction both in the greenhouse and under natural fields.

### Characterization of the selected PTGMS lines for fertility-sterility alternation in growth chambers

Uniform and healthy plants at the five-leaf stage were selected to transplant into plastic pots, and each pot contained five plants, labeled with plastic tags. Plant growth chambers (product model: HP1000GS; Wuhan Ruihua Instrumental Equipment Co., Ltd., China) were adjusted to the parameters before carrying out the experiments. Relative humidity, light duration, and daily mean temperature (DMT) were set according to the procedures described by Jiang (2015a). All plants were placed in plant growth chambers when the main panicle was at initiation stage for programmed temperature treatment lasting about 12 days (in the sensitive period), and then the plants were moved outside of the chambers, and they grew under the natural environment to heading. The pollen grains collected from the top five florets of each treated panicle during 5–12 days after the end of the treatment were observed under the microscope. Pollen sterility was recorded according to the I_2_-KI staining method (Virmani et al. 2003). The percentage of sterile pollen on average surpassed 99.5%; the PTGMS line was considered to be completely sterile under the treatment conditions.

### Dynamic observations of pollen fertility under natural field conditions

One hundred seeds of each line were sown at the experimental farm of Huazhong Agricultural University (HAU) under the conditions of different sowing date with a 15-day interval from 1April to 1 July during the summer season of 2018. Fifty uniform and healthy rice seedlings at the five-leaf stage were transplanted in fields with a spacing of 16.7 cm between plants and 26.7 cm between rows. At the initial heading stage, pollen grain samples of the top five spikelets of primary panicles were collected and then examined via the 1% I_2_-KI staining method (Virmani et al. 2003). Five randomly selected plants were investigated under the microscope dynamically every 2 days from 15 July to 5 October, as described by Jiang et al. (2015a). Daily mean temperature and daily minimum temperature (T_min_) data were recorded and provided by the Agricultural Meteorology Department of HAU.

### Evaluation of main agronomic traits and grain quality of the selected PTGMS lines and recurrent parent

The two selected PTGMS lines and recurrent parent were planted three times at 10-day intervals in the field during the winter season of 2017–2018 at the Rice Breeding Station of HAU in Lingshui County, Hainan Province, and seven times at 15-day intervals during the summer season of 2018 at the Experiment Farm of HAU in Wuhan City. Fifty plants of each material were transplanted in the field with a spacing of 16.7 cm between plants and 26.7 cm between rows. The heading date was recorded and, at maturity stage, five individual plants in the middle of the central row in each plot were taken for measurements of agronomic traits, including plant height, panicle number per plant, panicle length, and spikelets per panicle during the sterile period in the summer in Wuhan.Two traits, spikelet fertility and weight of 1000 grains, were added during the fertile period in the winter season in Hainan. Harvested bulk seeds from each plot in Hainan were used for analyzing rice quality, including brown rice percentage (%),milled rice percentage (%), head rice percentage (%), chalky rice percentage (%), chalkiness degree (%), rice grain length (mm), grain length/width ratio, alkali spreading value, amylose content (%),and gel consistency (mm). The *T*-test was used for examining the statistically significant differences between the selected lines and the recurrent parent.

### Evaluation of yield, main agronomic traits, and rice quality of hybrids in multi-location trials

The selected PTGMS lines and the recurrent parent were used as female parents to produce F_1_ seeds with two male parents, 9311and FXH No.1, which were the male parents of Fengliangyou4 and Fengliangyou Xiang 1, respectively. They were planted in a large area because of their high yield and good quality. The yield, main agronomic traits, and rice quality of the hybrids between the two selected PTGMS lines and two male parents were tested according to the method of the China national variety regional trial at six locations: Lingshui County of Hainan (18°32′N, 110°2′E), Experiment Farm of HAU (30°28′N, 114°20′E), Ezhou (30°23′N, 114°46′E), Jingzhou (30°12′N, 112°31′E), Xiaogan (30°54′N, 113°55′E), and Xiangyang (32°5′N, 112°8′E) of Hubei. Feng39S/9311 (Fengliangyou 4) and Feng39S/FXH No.1 (Fengliangyou Xiang 1) were used as control hybrids. The *T*-test was used to detect statistical differences between the selected hybrids and control hybrids.

## Additional files

Additional file 1: **Figure S1.** Analyzed results of genomics-based genetic background of BC_2_F_1_ plants by an SNP chip, RICE6K. The 12 chromosomes of rice are labeled 1 to 12. The blue lines indicate the introgressed segments of the recurrent parent. The red dots indicate the loci of the *Pi2* gene. The gray area indicates the different SNPs between recurrent and donor parents. The white area indicates the same genetic background between recurrent and donor parents. (TIF 8478 kb)

Additional file 2: **Figure S2.** Plant morphologies of the improved lines and the recipient parent in two ecological sites. (**a**) Plant morphologies of DB16206–34, DB16206–38, and Feng39S at sterile stage in Wuhan. (**b**) Plant morphologies of DB16206–34, DB16206–38, and Feng39S at fertile stage in Hainan. (TIF 5734 kb)

Additional file 3: **Figure S3.** Genomic differences between Hua1201S and Feng39S detected by an SNP chip, RICE6K. The 12 chromosomes of rice are labeled 1 to 12, and the red lines indicate the different SNPs between the recurrent and donor parents. (TIF 957 kb)

## Data Availability

All data generated or analyzed during this study are included in this published article [and its supplementary information files].

## Abbreviations

BB: Bacterial blight
BPH: Brown planthopper
CTP: Critical temperature point
MABC: Marker-assisted backcrossing
MAS: Marker-assisted selection
NILs: Near-isogenic lines
PTGMS: Photoperiod- and thermo-sensitive genic male sterile
QTL: Quantitative trait loci
R: Resistance
RFLP: Restriction fragment length polymorphism
RPGR: Recurrent parent genome recovery
SNP: Single nucleotide polymorphism
SSD: Stable sterility duration
SSR: Simple sequence repeat
