# Supplementary figures and images for: Improving rice blast resistance of Feng39S through molecular marker-assisted backcrossing
Source: Rice (N Y). 2019 Sep 9;12:70. doi: 10.1186/s12284-019-0329-3 (PMC6733936; doi:10.1186/s12284-019-0329-3)

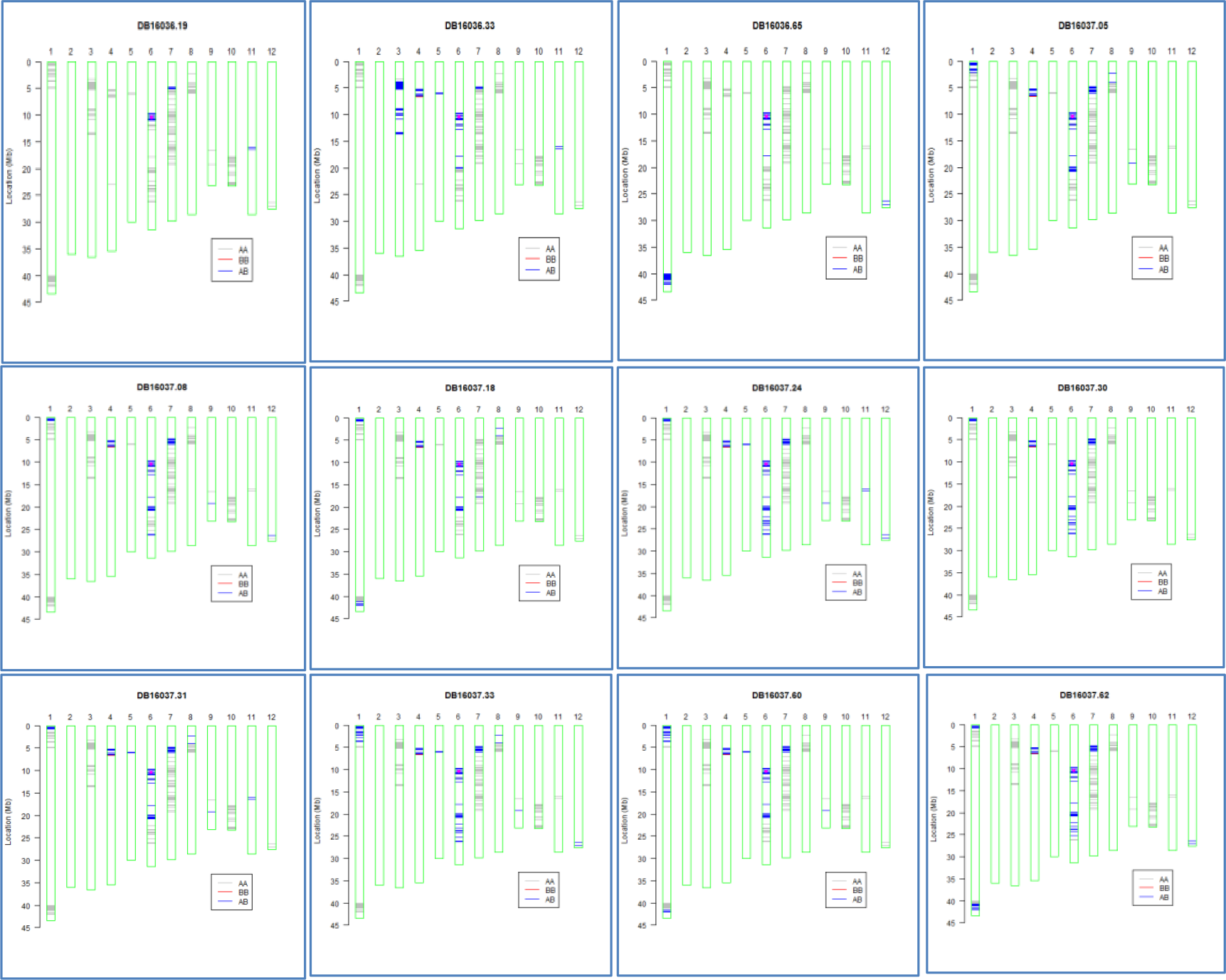

Supplement: Supplementary file 1 — Figure S1. Analyzed results of genomics-based genetic background of BC2F1 plants by an SNP chip, RICE6K. The 12 chromosomes of rice are labeled 1 to 12. The blue lines indicate the introgressed segments of the recurrent parent. The red dots indicate the loci of the Pi2 gene. The gray area indicates the different SNPs between recurrent and donor parents. The white area indicates the same genetic background between recurrent and donor parents. (TIF 8478 kb) [file 12284_2019_329_MOESM1_ESM.tif]

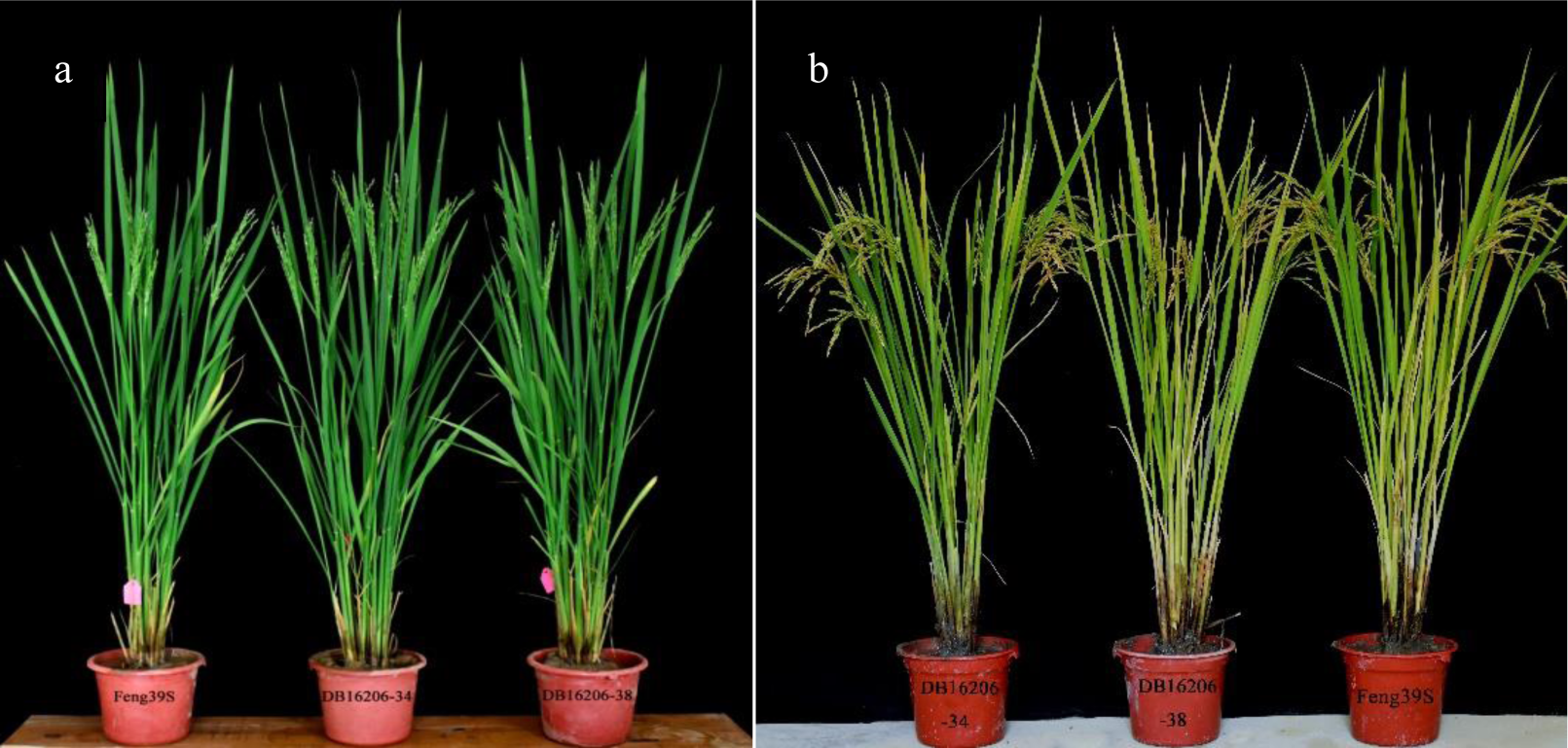

Supplement: Supplementary file 2 — Figure S2. Plant morphologies of the improved lines and the recipient parent in two ecological sites. (a) Plant morphologies of DB16206–34, DB16206–38, and Feng39S at sterile stage in Wuhan. (b) Plant morphologies of DB16206–34, DB16206–38, and Feng39S at fertile stage in Hainan. (TIF 5734 kb) [file 12284_2019_329_MOESM2_ESM.tif]

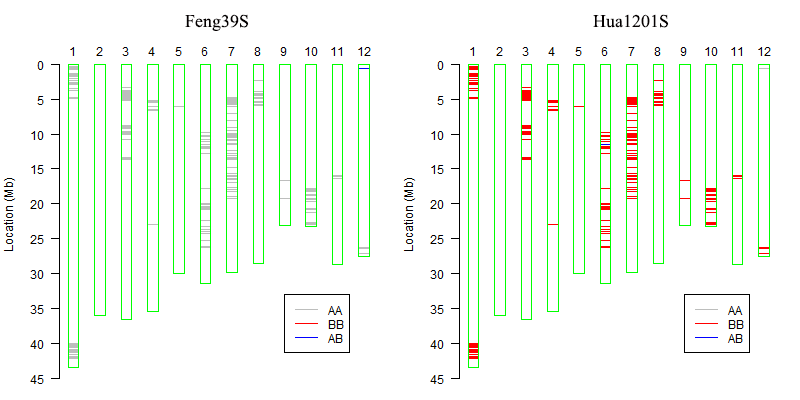

Supplement: Supplementary file 3 — Figure S3. Genomic differences between Hua1201S and Feng39S detected by an SNP chip, RICE6K. The 12 chromosomes of rice are labeled 1 to 12, and the red lines indicate the different SNPs between the recurrent and donor parents. (TIF 957 kb) [file 12284_2019_329_MOESM3_ESM.tif]
